# GPS Mobile Health Intervention Among People Experiencing Homelessness: Pre-Post Study

**DOI:** 10.2196/25553

**Published:** 2021-11-03

**Authors:** Leticia R Moczygemba, Whitney Thurman, Kyler Tormey, Anthony Hudzik, Lauren Welton-Arndt, Elizabeth Kim

**Affiliations:** 1 Health Outcomes Division College of Pharmacy University of Texas at Austin Austin, TX United States; 2 School of Nursing University of Texas at Austin Austin, TX United States

**Keywords:** GPS, mHealth, care coordination, people experiencing homelessness, homelessness, emergency department, health outcomes, health care costs, mobile phone

## Abstract

**Background:**

People experiencing homelessness are at risk for gaps in care after an emergency department (ED) or hospital visit, which leads to increased use, poor health outcomes, and high health care costs. Most people experiencing homelessness have a mobile phone of some type, which makes mobile health (mHealth) interventions a feasible way to connect a person experiencing homelessness with providers.

**Objective:**

This study aims to investigate the accuracy, acceptability, and preliminary outcomes of a GPS-enabled mHealth (GPS-mHealth) intervention designed to alert community health paramedics when people experiencing homelessness are in the ED or hospital.

**Methods:**

This study was a pre-post design with baseline and 4-month postenrollment assessments. People experiencing homelessness, taking at least 2 medications for chronic conditions, scoring at least 10 on the Patient Health Questionnaire-9, and having at least 2 ED or hospital visits in the previous 6 months were eligible. Participants were issued a study smartphone with a GPS app programmed to alert a community health paramedic when a participant entered an ED or hospital. For each alert, community health paramedics followed up via telephone to assess care coordination needs. Participants also received a daily email to assess medication adherence. GPS alerts were compared with ED and hospital data from the local health information exchange (HIE) to assess accuracy. Paired *t* tests compared scores on the Patient Health Questionnaire-9, Medical Outcomes Study Social Support Survey, and Adherence Starts with Knowledge-12 adherence survey at baseline and exit. Semistructured exit interviews examined the perceptions and benefits of the intervention.

**Results:**

In total, 30 participants were enrolled; the mean age was 44.1 (SD 9.7) years. Most participants were male (20/30, 67%), White (17/30, 57%), and not working (19/30, 63%). Only 19% (3/16) of the ED or hospital visit alerts aligned with HIE data, mainly because of patients not having the smartphone with them during the visit, the smartphone being off, and gaps in GPS technology. There was a significant difference in depressive symptoms between baseline (mean 16.9, SD 5.8) and exit (mean 12.7, SD 8.2; *t*_19_=2.9; *P*=.009) and a significant difference in adherence barriers between baseline (mean 2.4, SD 1.4) and exit (mean 1.5, SD 1.5; *t*_17_=2.47; *P*=.03). Participants agreed that the app was easy to use (mean 4.4/5, SD 1.0, with 5=strongly agree), and the email helped them remember to take their medications (mean 4.6/5, SD 0.6). Qualitative data indicated that unlimited smartphone access allowed participants to meet social needs and maintain contact with case managers, health care providers, family, and friends.

**Conclusions:**

mHealth interventions are acceptable to people experiencing homelessness. HIE data provided more accurate ED and hospital visit information; however, unlimited access to reliable communication provided benefits to participants beyond the study purpose of improving care coordination.

## Introduction

### Background

Mobile phone ownership is nearly ubiquitous among American adults, with 96% owning a mobile phone of some type, and most (81%) mobile phones are smartphones [1]. Accordingly, mobile technology is increasingly common in the health care sector. Mobile devices are being used for medical diagnostics [2], disease monitoring [3], smoking cessation [4], and dietary tracking [5]. Smartphone capabilities, including texting and apps, have contributed to improved medication adherence [6], higher attendance at medical appointments [7], and increased vaccination rates [8]. Mobile technology has also been explored as a useful tool to bolster the transmission of information and care coordination during transitions of care [9,10], and studies have demonstrated the potential of mobile technology to improve communication among health care providers and populations at risk for poor outcomes, including people of lower socioeconomic status [11,12].

Recent estimates of mobile phone use among the homeless population indicate that 89% of the people report having and using a mobile phone [13], and researchers have begun to explore the possibility of using mobile technology to improve the health of people experiencing homelessness. For example, Burda et al [14] concluded that mobile phones are a feasible way to monitor and manage medication regimens for people experiencing homelessness with co-occurring disorders. Furthermore, in a survey of people experiencing homelessness, 77% of the respondents were interested in appointment reminders, and most were interested in medication refill reminders (66%) and medication taking reminders (60%) [13]. Despite the accumulating evidence that mobile health (mHealth) interventions among homeless populations are feasible, GPS-enabled mHealth (GPS-mHealth) interventions in this population have remained underexplored. The purpose of this study, therefore, is to investigate the acceptability and preliminary outcomes of a GPS-mHealth intervention designed to improve care coordination in a sample of people experiencing homelessness.

Evidence suggests that the health service experiences of people experiencing homelessness are often interrupted and involve extensive barriers, including unmet physical needs, lack of affordable and available services, and lack of compassion that prevents people experiencing homelessness from accessing appropriate community-based services [15-17]. These barriers lead to disruptions in continuity of care, which is problematic because of evidence that continuity of care—that is, timely, accessible, person-centered, and coordinated care—improves outcomes [18]. Interventions such as case management, respite care, and housing services that target critical transition points have led to decreased acute care use [19] in people experiencing homelessness. Community paramedics have also been used to coordinate care and link high-risk patients to needed health and social services [20], which has led to reduced health care use among diverse populations and improvements in patient outcomes [21]. Despite these multifaceted programs, interventions, and service delivery models intended to improve care coordination among people experiencing homelessness, gaps in services along the continuum of care persist.

### Study Premise and Objectives

This study focuses on the significant gap along the continuum of care that begins at the point of an emergency department (ED) visit or hospitalization for people experiencing homelessness. The study intervention was created on the basis of feedback from health care providers and case managers who deliver care to homeless individuals, and the fact that fragmented communication among various health care organizations limits the ability to provide real-time information about ED or hospital visits. When a person experiencing homelessness enters the ED or hospital, they are at high risk of losing contact with community-based health care providers and case managers [22]. This is exacerbated in the people experiencing homelessness living with depression as it is more difficult to manage their chronic conditions, including attending appointments and taking medications as prescribed [23]. The loss of contact between homeless individuals and their community-based care team creates a time of high risk for the individual and represents missed opportunities to provide services and potentially decrease acute health care use. For preventing or minimizing this loss of contact, this study used geofencing to create virtual boundaries that triggered automatic notification of community paramedics if and when a person experiencing homelessness visited an ED or hospital. The use of such geofencing technology in health care has been previously studied in smoking cessation, dietary recommendations, anxiety, and hospitalizations in patients with cardiovascular disease [5,10,24,25]. However, the utility of a GPS-mHealth intervention specifically in transitions of care for people experiencing homelessness has not been previously reported. Therefore, the following research questions guided this study:

1. What is the accuracy of GPS technology in terms of tracking participant visits to the ED or hospital?
2. How do depression symptoms, medication adherence, social support, and experience with and perceptions of GPS and mobile phone technology compare at baseline and exit?
3. What is the number and type of community health paramedic encounters?
4. What concerns do participants express regarding technology or privacy?

## Methods

### Design and Participants

This study used a pre-post design with assessments at baseline, 1 month, 2 months, 3 months, and 4 months after enrollment to evaluate the acceptability and preliminary outcomes of a GPS-enabled mHealth intervention. Participants were recruited from 2 churches that provided services to people experiencing homelessness. The first serves breakfast at 5:45 AM two mornings each week and is open to anyone in the community. The research staff attended this breakfast once per week for the study duration. Potential participants were referred to study staff for eligibility screening by either the meal program coordinator or the police officer assigned to the downtown Homeless Outreach Service team, whose job function includes attending these twice weekly breakfasts. The second church site doubles as a navigation center for people experiencing homelessness during weekdays. Services at the navigation center include coordinated assessments for housing, assistance with obtaining IDs, and case management. The research staff were on site at the navigation center 2 to 3 days per week for the study duration. Similar to the first site, potential participants were referred by the director of the navigation center or by navigation center volunteers to study staff for study eligibility screening.

Recruitment occurred between October 2018 and April 2019. Community partners assisted with recruitment by distributing flyers to clients and by referring potential participants to research staff. Participants also referred peers who were potentially eligible to the study staff. Potential participants were screened for study eligibility on site at the churches by a member of the research team. The eligibility criteria included (1) being at least 18 years old, (2) currently experiencing homelessness defined as where the person had slept most nights in the past 30 days, (3) score of at least 10 on the Patient Health Questionnaire-9 (PHQ-9), (4) currently prescribed at least 2 medications for chronic medical conditions, (5) diagnosed with at least 1 chronic medical condition, and (6) experienced at least 2 hospitalizations or ED visits in the past 6 months. Exclusion criteria included (1) onset in the past 3 months of depressive symptoms and (2) suicide attempts or suicidal ideation in the past 6 months.

This study was approved by the institutional review board of the university. Individuals interested in participating were screened by research staff, and, if eligible to continue, study details, including the purpose, procedures, risks, and benefits of study participation, were explained. If participants remained interested, informed consent was obtained. None of the participants who were eligible for the study declined to participate after being informed of the study details. After obtaining informed consent, a researcher administered a series of baseline assessments to collect information about demographics, health history, medication adherence, social support, and recent ED visit and hospitalizations. After completion of the surveys, participants were provided with a smartphone activated with a plan for unlimited texting, calling, and data; a hard-plastic smartphone case; and an armband to use for securing the smartphone. Participants were also given US $25 cash for the time spent enrolling in the study and a 31-day unlimited use bus pass to ensure their ability to attend the monthly follow-up assessment. They then received training on the intervention. The training described the expectations of participants, including keeping track of the smartphone, keeping it turned on and charged, attending monthly check-in visits, answering the daily email regarding medication adherence, and responding to community health paramedics or research staff as applicable. The training also included how to use the smartphone, set up voicemail, access email and SMS text messages, and access and use the bus pass. Participants were also informed that one replacement smartphone would be issued if their study smartphone was lost, stolen, or broken during the 4-month study.

### GPS-mHealth Intervention

For this study, a mobile app was used to establish and monitor geofences around the 10 EDs located within the city limits where this study took place. The geofences were established using the mobile app so that when a participant entered a local ED or hospital, the research staff and the commander of the community paramedic team would receive an email notification. The email notification sent a secure link to view the participant’s name, geofence location, date, and time of entry and exit. On receipt, the commander tasked a community health paramedic member of his team to contact the participant via their smartphone within 2 business days of the geofence entry to follow-up on the visit and any identified health or social needs. The community health paramedic completed an event form documenting the participant-reported reason for the hospital or ED visit, admission, and discharge dates; if the ED or hospital visit was potentially preventable; what intervention may have prevented the ED and hospital visit; and the duration of the community health paramedic visit with the participant.

In addition to the geofencing and care provided by community health paramedics as needed, the intervention had two additional components: (1) monthly in-person meetings and (2) daily adherence reminder emails. In-person meetings occurred between each participant and research staff at enrollment; 1-, 2-, and 3-month follow-up appointments; and at the exit. Monthly follow-up visits (at 1, 2, and 3 months) were scheduled to maintain contact with participants and to identify any issues with the technology. Participants were also asked at these monthly meetings if they had visited the ED or been hospitalized in the past 30 days. At months 1, 2, and 3, participants received US $10 cash and an additional 31-day bus pass. Next, participants received an email every evening at 8 PM asking if they had taken their medications that day. Response options were “yes” or “no,” with a follow-up question requesting a short reason why they had not taken their medication if applicable. During the exit interviews, participants responded to a series of questionnaires before engaging in a semistructured interview to assess the overall acceptance of the intervention. Textbox 1 summarizes the interview guidelines. Finally, the local health information exchange (HIE) provided research staff with dates of hospital admissions and ED visits, as applicable, for participants during the 4-month study period.

**Semistructured interview guide.**

**Questions about the study**

1. Please describe your experience with this research study (probe 1: Did you experience benefits from participating in this study? probe 2: Was participating in this study helpful to you? probe 3: Were there any difficulties that you experienced with this study?)
2. Can you share any barriers to study participation that you experienced? (Examples may include keeping the smartphone secure or charged)
3. What strategies did you use to successfully complete the study requirements? (This includes things such as keeping the smartphone charged and operational as well as attendance at monthly check-in visits)
4. What concerns did you have about your visits to the emergency room and hospital being monitored with GPS technology?
5. Can you describe any experiences or interactions you had with community health paramedics?
6. What suggestions do you have for us to improve this intervention for people in the future?

### Measurements

#### Sociodemographic and Health-Related Variables

At baseline, sociodemographic characteristics, including sex, race, highest education obtained, veteran status, and income, were collected. Participants were also asked a series of six questions from the American Community Survey designed to identify individuals who may experience functional limitations [26]. Response options were 1=yes or 0=no. The items were summed for a total score, with higher scores indicating a higher burden of functional limitations. The Cut down, Annoyed, Guilty, and Eye-opener questionnaire, a 4-item screening tool, was used to screen for alcohol use [27]. Response options were 1=yes or 0=no. The items were summed for a total score, and a total score of >2 was considered clinically significant [27]. The single-item screen in which the participant is asked, “how many times in the past year have you used an illicit drug or used a prescription medication for nonmedical reasons?” was used to screen for substance use [28]. Responses ≥1 were considered to be positive.

#### Health Literacy

Health literacy was measured using the Brief Health Literacy Screening Tool [29], which comprises 4 questions that assess respondents’ ability to complete tasks such as filling out medical forms, reading hospital paperwork, and learning about one’s medical condition. Each item is worth 1 to 5 points, depending on the response. Scores were summed for a composite score ranging from 4 to 20. Scores of 4-12 indicate limited health literacy, scores of 13-16 indicate marginal health literacy, and scores of 17-20 indicate adequate health literacy [30].

#### Accuracy of the GPS Technology

The accuracy of the GPS technology was measured in 2 ways. First, when community health paramedics received an alert indicating that a participant had entered a geofence at an area hospital, a community health paramedic attempted to make contact with the participant within 2 business days. If contact was established, the community health paramedic confirmed the visit to the ED and hospital, as indicated by the geofence alert. Second, at the end of the study, the research staff obtained use records from the HIE. These records provided the dates of participants’ ED and hospital visits during the study period. Use records for the 25 participants for whom HIE data were collected were triangulated with geofence entry notifications to measure the accuracy of the GPS technology.

#### Depression

The 9-item PHQ-9 was used to establish participant eligibility and as a baseline measure for depression symptoms. The PHQ-9 is a reliable and valid tool for diagnosing and grading depressive symptom severity [31]. Each item is scored from 0-3 and then summed. Scores of 5, 10, 15, and 20 represent cutoff points for mild, moderate, moderately severe, and severe depression, respectively [31]. To be eligible to participate in this study, individuals were required to score at least 10, indicating moderate depression.

#### Medication Adherence

Medication adherence was measured using a modified version of the Adherence Starts with Knowledge-12 (ASK-12). The ASK-12 is a brief, 12-item scale with 3 subscales that measure medication behavior, health beliefs, and inconvenience/ forgetfulness [32]. For this study, we modified the subset of 5 questions assessing medication behavior into dichotomous yes/no response options to assess medication adherence during the preceding month. The number of yes responses was counted and summed for a medication behavior subscale score. Scores on the full ASK-12, with the modified medication behavior subscale, ranged from 12-40, with higher scores indicating greater barriers to adherence. At baseline, the full scale with the modified behavior subscale was used. At monthly visits and exit, only the modified medication behavior subscale was used as it was unlikely that medication beliefs would change within the short time frame of this study.

#### Social Support

Social support was measured using the Medical Outcomes Study Social Support Survey, a valid and reliable tool that has been used in multiple groups across various conditions [33]. It includes 19 questions yielding four subscales—emotional/ informational support, tangible support, affectionate support, and positive social interaction. Each item is rated using a Likert scale ranging from 1 (none of the time) to 5 (all of the time). The total score was calculated by summing all 19 questions and averaging them. Higher scores represent greater levels of social support.

#### Experience With and Acceptance of Technology

At baseline, experience with mobile phone technology was measured using a series of questions asking about current mobile phone ownership, mobile phone service, length of time owning a mobile phone, ability to charge the mobile phone, and if the participant had had a mobile phone stolen before. Acceptance of technology was measured at baseline and exit. At baseline, acceptance of technology was measured using a modification of the Technology Acceptance Questionnaire [34]. At baseline, 17 items were used, and at exit, a subset of these items, as well as an additional 8 items, were used. Each item is rated using a Likert scale ranging from 1=strongly disagree to 5=strongly agree. Higher scores indicate greater acceptance of technology. In addition, at exit, participants were asked how often they were able to charge their smartphone with options ranging from “None of the time” to “Always.”

#### Quality of Care Transitions

Self-reported ED and hospital use were assessed at baseline, monthly visits, and exit. If participants indicated that they had visited the ED or hospital within the past month, their experience and perception of patient-centeredness of their care were assessed using the care transitions measure (CTM), a 15-item measure reflecting 4 content domains [35]. The domains include critical understanding, important preferences, management preparation, and care plans [35]. Participants used a 5-point Likert scale ranging from 1 (strongly disagree) to 5 (strongly agree) to rate the quality of various components of a care transition within each domain. Lower scores indicate a poorer quality transition, and higher scores indicate a better transition. The CTM was administered at each monthly visit, during which a participant reported an ED or hospital visit. If someone reported more than 1 visit in the previous month, the CTM was completed only for the most recent visit.

### Data Analysis

All statistical analyses were performed using IBM SPSS Statistics for Windows (version 25.0, IBM Corp). Descriptive statistics were used to describe the sociodemographic and health characteristics of the sample and all study measures. Accuracy of the geofence entry notifications was determined by calculating the percentage of notifications that aligned with HIE use data. Paired sample *t* tests were used to compare scores at baseline and exit on the PHQ-9, ASK-12, Medical Outcomes Study, and technology acceptance scales.

Qualitative content analysis was used to identify participants’ acceptance of the intervention. All interviews were audio recorded and transcribed verbatim to facilitate coding and analysis. After a thorough reading and deductive coding of 5 representative transcripts by 2 members of the study team (LRM and WT), a consensus meeting was held to discuss and agree upon the codes. Discrepancies were resolved by discussing the context for each phrase being analyzed. After the meeting, a codebook was developed. The remaining interviews were divided between the 2 authors and coded separately. After coding was complete, the study team organized the codes into categories.

## Results

### Overview

Between October 2018 and April 2019, research staff screened 39 individuals for participation; of the 39 individuals, 32 (82%) met the eligibility criteria, and 30 (77%) were enrolled in the study. The 2 individuals who were eligible to participate but did not enroll did not return for the subsequent enrollment visit in the study after screening. The reasons for ineligibility for the study were not scoring at least 10 on the PHQ-9 (2/39, 5%), not having been to the ED or hospital at least twice in the past 2 months (2/39, 5%), not being prescribed a medication (2/39, 5%), and endorsing suicidal ideation (1/39, 3%). The participant who endorsed suicidal ideation was referred to the public safety officer on site at the community entity for appropriate follow-up and mental health services. Of the 30 participants, 10 (33%) were screened and enrolled at the first church with 2 weekly breakfasts, and the remaining 20 (67%) were screened and enrolled at the navigation center housed in a church.

Of the 30 participants enrolled, 19 (63%) completed the 4-month intervention, with a completion rate of 63%. Of the 11 participants who did not complete the intervention, 6 (55%) were withdrawn from the study after they reported their second smartphone lost or stolen, 2 (18%) notified the research staff that they were moving to a different town, 2 (18%) were lost to follow-up, and 1 (9%) voluntarily withdrew from the study after losing his first smartphone. Of these 11 participants, 4 (36%) completed all but the exit data collection.

### Quantitative Results

#### Participant Demographics and Health-Related Characteristics

Participants comprised 30 people experiencing homelessness. On average, participants were male (20/30, 67%), aged 44.1 years (SD 9.7 years), White (17/30, 57%), never married (17/30, 57%), and not working because of disability or other medical reasons (19/30, 63%). At baseline, participants reported a mean of 2.8 (SD 1.4) chronic conditions, and most (26/30, 87%) experienced multiple chronic conditions. All participants were prescribed at least 2 medications at baseline; 53% (16/30) were prescribed 4 or more medications. Tables 1 and 2 provide a summary of demographic and health-related characteristics.

**Table 1:** Summary of demographic information (N=30).

Variables				Values
**Age (years)**				
	Mean (SD)		44.1 (9.7)	
	Median		46	
**Gender, n (%)**				
	Male		20 (67)	
	Female		8 (27)	
	Transgender female		1 (3)	
	Other		1 (3)	
**Self-reported race or ethnicity, n (%)**				
	White		17 (57)	
	Black or African American		7 (23)	
	Hispanic		2 (7)	
	Native American		1 (3)	
	Other		3 (10)	
**Marital status, n (%)**				
	Married or domestic partnership		4 (13)	
	Divorced		9 (30)	
	Single or never married		17 (57)	
**Children, n (%)**				
	Yes		18 (60)	
	**Number of children for those with ≥ 1 child**			
		Mean (SD)	2.9 (1.6)	
		Median	2	
**Highest level of education, n (%)**				
	Less than high school		8 (27)	
	High school graduate or GED^a^		12 (40)	
	Trade, technical, or vocational training		4 (13)	
	Some college		5 (17)	
	Other		1 (3)	
**Military veteran, n (%)**				
	Yes		2 (7)	
**Employment status^b^, n (%)**				
	Not employed		24 (83)	
	Employed		5 (17)	
**Reason if unemployed^b,c^, n (%)**				
	Looking for work		6 (23)	
	Laid off		2 (8)	
	Disabled or medical reason		19 (73)	
	Other		3 (12)	
**Annual income (US $), n (%)**				
	0-10,000		27 (90)	
	10,001-20,000		3 (10)	
**Slept most nights^c^, n (%)**				
	On the street		20 (67)	
	In a shelter		2 (7)	
	Other		9 (30)	
**Length of homelessness (years)**				
	Mean (SD)		8.1 (7.7)	
	Median		5	

^a^GED: general educational development.

^b^Data were missing for some participants.

^c^Respondents may have chosen more than one response.

**Table 2:** Summary of baseline health information (N=30)^a^.

Variables		Values
**Number of chronic conditions**		
	Mean (SD)	2.8 (1.4)
	Hypertension, n (%)	19 (63)
	Diabetes mellitus, n (%)	5 (17)
	High cholesterol, n (%)	9 (30)
	Asthma, n (%)	12 (40)
	Chronic obstructive pulmonary disease, n (%)	11 (37)
	Congestive heart failure, n (%)	2 (7)
**Number of prescribed medications, n (%)**		
	2-3	14 (47)
	4-5	12 (40)
	≥6	4 (13)
**Self-reported number of ED^b^ visits or hospitalizations in past 6 months, n (%)**		
	2	18 (60)
	3	6 (20)
	4	3 (10)
	≥5	3 (10)
**Visited ED in past 30 days (self-report), n (%)**		
	Yes	17 (57)
**Visited hospital in past 30 days (self-report), n (%)**		
	Yes	7 (23)
**Functional limitations, n (%)**		
	Deaf or difficulty hearing (yes)	8 (27)
	Blind or difficulty seeing when wearing glasses (yes)	11 (37)
	Difficulty walking or climbing stairs (yes)	15 (50)
	Difficulty dressing or bathing (yes)	5 (17)
**Number of functional limitations, n (%)**		
	1	8 (27)
	2	6 (20)
	≥3	6 (20)
**CAGE^c^ substance abuse screening score, n (%)**		
	≥2	10 (33)
**Drug use in past year, n (%)**		
	Yes	16 (53)
**Health literacy level**		
	Mean (SD)	13.7 (5.2)
	Median	14.5
	Limited, n (%)	13 (43)
	Marginal, n (%)	5 (17)
	Adequate, n (%)	12 (40)

^a^Percentages are out of 30 and more than one response was allowed per respondent.

^b^ED: emergency department.

^c^CAGE: Cut down, Annoyed, Guilty, and Eye-opener.

#### Accuracy of the GPS Technology

Accuracy of the GPS technology was calculated for the 25 participants who completed a release of information form, giving permission for the research team to access data in the HIE. During the 4-month study period, HIE use data indicated that these participants made 16 hospital or ED visits. Community health paramedics received 14 total geofence entry notifications during the study period; of these 14 notifications, 2 (14%) were from participants without a release of information for the HIE. Thus, community health paramedics received 12 geofence entry notifications for the 25 participants from whom HIE data were available. However, only 3 of the geofence entry notifications were consistent with the HIE use data for an overall accuracy rate of 19%.

Of the 16 ED and hospital visits reported by the HIE data for which community health paramedics did not receive geofence entry notifications, 4 (25%) occurred during the first month of the intervention, a time during which the research staff identified a technical issue with the mobile app and geofence entries were not being received. Of these 16 visits, 3 (19%) occurred in the window of time during which the participant was without the study-assigned smartphone as the smartphone had been stolen or misplaced but not yet replaced. It is unclear why the remaining 43% (6/14) ED and hospital visits reported by the HIE data did not result in a geofence notification entry.

#### Community Health Paramedic Interventions

Community health paramedics successfully reached participants to conduct follow-up and provide care coordination assistance after 79% (11/14) of geofence notifications. Of these 11 contacts, 10 (91%) lasted ≤10 minutes, and 1 (9%) contact lasted between 11 and 20 minutes. Of these 11 contacts, 3 (27%) participants reported having accompanied a friend or family member to the ED and were not seen themselves, and 1 (9%) participant reported having visited the hospital campus for a scheduled medical visit. Thus, 36% (4/11) of these geofence notifications were classified as false positives. Of the remaining 7 contacts, 3 (43%) aligned with the HIE notification data. Of the remaining 4 contacts, 3 (75%) did not align with the HIE data, as the participants did not have a release of information form on the file. It is unclear why the remaining contact did not register with the HIE.

Community health paramedics determined that 43% (3/7) of the ED visits were emergent and likely unavoidable. Reasons for the emergent ED visits included chronic pulmonary obstructive disease exacerbation, a physical altercation at a local shelter, and uncontrolled epigastric pain. Reasons for the remaining ED visits were skin irritation because of scabies infection, shoulder pain, and 2 visits for gastrointestinal illness. Community health paramedics judged each of these 4 visits to be due to ambulatory care–sensitive conditions that could have been appropriately managed in the outpatient setting.

#### Depression

There was a significant difference in depressive symptoms between baseline (mean 16.9, SD 5.8) and exit (mean 12.7, SD 8.2; *t*_19_=2.892; *P*=.009), indicating fewer depressive symptoms at the 4-month exit.

#### Medication Adherence

At baseline, scores on the ASK-12 ranged from 14-30 (mean 20.5, SD 4.4). Among those who completed the 4-month intervention, there was a significant difference in medication behavior between baseline (mean 2.4, SD 1.4) and exit (mean 1.5, SD 1.5; *t*_17_=2.47; *P*=.03), indicating that at the 4-month exit visit, there were fewer barriers to taking medications.

#### Social Support

There was no significant difference in social support between baseline (mean 3.2, SD 1.1) and exit (mean 2.9, SD 1.3; *t*_18_=1.25; *P*=.23).

#### Experience With and Acceptance of Technology

At baseline, 50% (15/30) of participants reported having a mobile phone. Of these 15 patients, 12 (80%) had current wireless service (4/12, 33% participants had pay as you go service plans; 3/12, 25% had prepaid plans, 3/12, 25% had month-to-month contracts; and 2/12, 17% had free minutes through government-funded plans). Of the 15 participants with mobile phones, 13 (87%) reported that their mobile phones could support both SMS text messaging and mobile apps. At the exit interview, participants agreed that the smartphone app was easy to use (mean 4.4, SD 1.0), that they had the knowledge to use the smartphone app (mean 4.6, SD 0.5), and that they planned to continue using both a smartphone (mean 4.5, SD 0.6) and GPS technology (mean 4.4, SD 0.5). The acceptance of technology questionnaire indicated that participants had a high level of agreement at baseline and exit with items such as having the resources and knowledge to use smartphone technology and being comfortable with the health care team being alerted about ED or hospital use. There was a significant increase in agreement level from baseline (mean 3.9, SD 0.8) to exit (mean 4.4, SD 0.5) for the item, “My friends would encourage me to use this Smartphone app.” Participants’ agreement level increased for several other items, such as having the knowledge and resources to use GPS technology from baseline to exit, but not significantly. Table 3 summarizes the participants’ technology acceptance at baseline and the 4-month exit interview.

**Table 3:** Perceptions of acceptance of technology at baseline and 4-month exit interview.

Item	Baseline, mean (SD)	Exit, mean (SD)	*P* value
I have the resources necessary to use smartphone technology	4.4 (0.5)	4.4 (1.0)	.86
I have the knowledge necessary to use smartphone technology	4.6 (0.4)	4.7 (0.5)	.28
I can get help from others when I have difficulties using smartphone technology	4.2 (0.7)	4.5 (0.8)	.28
I find GPS technology useful in my daily life	4.2 (0.8)	4.4 (1.0)	.39
I find GPS technology easy to use	4.1 (0.8)	4.3 (1.1)	.33
I have the resources necessary to use GPS technology	3.8 (1.2)	4.2 (1.3)	.29
I have the knowledge necessary to use GPS technology	4.1 (1.0)	4.6 (0.5)	.06
I am comfortable with my health data being stored online	3.6 (1.3)	4.1 (0.9)	.13
I believe my health information will be protected on a smartphone	3.7 (1.2)	3.6 (1.1)	.88
I am comfortable with my health care team being alerted when I go to the emergency department or hospital	4.8 (0.4)	4.7 (0.5)	.33
I think using GPS is a good way to notify my health care team when I visit the emergency department or hospital	4.5 (0.6)	4.7 (0.5)	.27
I think using this smartphone app can help me improve my overall health	4.3 (0.6)	4.5 (0.5)	.16
My friends would encourage me to use this smartphone app	3.9 (0.8)	4.4 (0.5)	.04
My family members would encourage me to use this smartphone app	4.0 (1.0)	4.1 (1.0)	.85

#### Quality of Care Transitions

At baseline, 57% (17/30) of participants self-reported at least one ED or hospital visit in the previous 30 days and completed the CTM-15. At months 1, 2, 3, and exit, 33% (10/30), 13% (4/30), 13% (4/30), and 17% (5/30) of participants, respectively, self-reported at least 1 ED or hospital visit in the previous 30 days and completed the CTM-15 for their most recent visit. The mean score for the critical understanding and management preparation domains was 4.1, indicating that participants generally agreed that they left the hospital or ED understanding how to manage medications and their health. The mean score for the preferences important domain was 4.0 (SD 0.1), which means that participants agreed that hospital staff took their preferences for health care needs into account when planning for discharge. The lowest level of agreement was with the care plan domain (mean 3.8, SD 0.0). Table 4 provides a summary of the scores for each item and domain.

**Table 4:** Summary of perceptions of the quality-of-care transitions using the care transitions measure (N=40 hospital or ED^a^ visits).

Domains and items		Mean (SD)^b^
**Critical understanding**		
	When I left the hospital or ED, I clearly understood the purpose for taking each of my medications.	4.1 (0.8)
	When I left the hospital or ED, I clearly understood how to take each of my medications, including how much I should take and when.	4.3 (0.7)
	When I left the hospital or ED, I clearly understood the possible side effects of each of my medications.	4.0 (1.1)
	When I left the hospital or ED, I had a good understanding of the things I was responsible for in managing my health.	4.2 (0.8)
	When I left the hospital or ED, I was confident that I knew what to do to manage my health.	3.9 (1.0)
	When I left the hospital or ED, I was confident I could actually do the things I needed to do to take care of my health.	3.8 (1.1)
	Domain overall mean	4.1 (0.2)
**Preferences important**		
	Before I left the hospital or ED, the staff and I agreed about clear health goals for me and how those would be reached.	3.9 (1.2)
	The hospital staff took my preferences into account in deciding what my health care needs would be when I left the hospital or ED.	4.1 (1.0)
	The hospital staff took my preferences into account in deciding where my health care needs would be met when I left the hospital or ED.	4.0 (1.1)
	Domain overall mean	4.0 (0.1)
**Management preparation**		
	When I left the hospital or ED, I had all the information I needed to be able to take care of myself.	4.0 (0.9)
	When I left the hospital or ED, I clearly understood how to manage my health.	3.9 (0.9)
	When I left the hospital or ED, I clearly understood the warning signs and symptoms I should watch for to monitor my health condition.	4.2 (0.8)
	When I left the hospital or ED, I had a good understanding of my health condition and what makes it better or worse.	4.1 (0.9)
	Domain overall mean	4.1 (0.1)
**Care plan**		
	When I left the hospital or ED, I had a readable and easily understood written list of appointments I needed to complete within the next several weeks.	3.8 (1.1)
	When I left the hospital or ED, I had a readable and easily understood written plan that described how all of my health care needs were going to be met.	3.8 (1.2)
	Domain overall mean	3.8 (0.0)

^a^ED: emergency department.

^b^Participants indicated their level of agreement with each item using a Likert scale from 1=strongly disagree to 5=strongly agree.

### Qualitative Findings

Of the 30 participants, 17 (57%) completed an exit interview. During data analysis, the first 2 authors of this study organized the codes into the following categories: (1) benefits of study participation, (2) challenges to study participation, (3) perceptions of GPS technology, and (4) suggestions for improvement.

Overall, participants reported positive experiences with study participation. They also identified several benefits, defined as any real or perceived aid or assistance from participating in the research study or having access to the unlimited use of a smartphone. Benefits included self-management support, improved social connections, and improved well-being. An example of how study participation provided self-management support is demonstrated by this quote:

[...] there was a time when I [...] would be confused as to whether or not I took my medicine. Sometimes I would go days without even thinking about it, you know? But now, I am confident knowing that every morning you know “Bam!”, you know it’s [daily email] right there and I had my medication and had taken it. There was never any more confusion.

Social connections were facilitated by the ability to call friends and family, to stay up to date on current events by reading the news on the internet, and to use social media sites. Several participants described using the smartphone to reconnect with the family from out of state. One participant put it succinctly as follows:

[...] being homeless, you can be very bored sometimes with nothing to do. And, [with the phone] I had something to do. You can read the news and find out what’s going on in the world. Or, you know, keep in touch with my friends with email.

Participants also described improved well-being as they did not have to worry about paying for their smartphone, were able to travel to appointments because of bus pass on the smartphone, and felt more secure in their environments with the ability to contact the police or emergency medical services in the case of an emergency. An example of how study participation improved well-being is demonstrated by the following quote:

It was a godsend. It really was, I mean because I didn’t have to worry about a lot of things. I could make phone calls when I needed to. It just took a lot of burden off me, knowing that I had a bus pass. I had a phone I could use you know if I got in trouble or something or was in a bad situation.

Challenges to study participation were defined as circumstances in which participants had to navigate to access, use, and benefit from services and resources, including the research study itself. Challenges included differential treatment because of homelessness, difficulty with technology, and keeping the smartphone secure. For example, differential treatment resulted in participants having difficulty keeping their smartphones charged as business owners do not allow people experiencing homelessness to spend time charging smartphones in their establishments. Some participants also described trouble with technology, such as short battery life and slow internet service. Finally, keeping the smartphone secure required constant vigilance on the part of participants, and even with creative solutions for safekeeping, many experienced theft or damage to their smartphones. One participant expressed his desire for smartphones to be replaced up to 4 times, saying as follows:

[...] the fact is, anything can happen out here. Like you know, I was charging my phone at Starbucks. I fell asleep, and when I woke up, my phone was stolen. Got my second phone…but I forgot to put the case back on and water hits it and its out.

Perceptions of the GPS technology were uniformly positive, as each participant who completed the exit interview denied having concerns about the community health paramedics or research staff knowing when they visited the ED or hospital. One participant clearly articulated this by saying the following:

...you know, that kind of thing right now is the least of my concerns. If you’re sleeping in an alley or somewhere else, you’re not really worried about somebody knowing that you’ve been to the hospital, or at least I’m not.

Suggestions for improvement included two main subcategories: helping to complete the study requirements and tailoring the intervention. Participants suggested more teaching about using the smartphone and its functions and providing portable battery chargers to help overcome some of the technical challenges to study completion that participants faced. Participants also suggested sending daily messages via text instead of email and indicated that personalized and tailored medication reminders for their individual medication regimens would be helpful.

## Discussion

### Principal Findings

The results of this study contribute to a small but growing body of literature documenting the utility of mHealth interventions among people experiencing homelessness. First, our findings suggest that GPS technology is not a reliable method for tracking visits to the ED or hospital among people experiencing homelessness. The geofence notifications aligned with objective HIE use data only 18.8% of the time, indicating that the community health paramedics were unable to connect with participants to provide follow-up assistance with care coordination after most participant ED and hospital visits. This finding was surprising given recent evidence that a smartphone app used by 12 patients with low income had 75% accuracy in detecting real-time ED or hospital use over a 3-month period [36]. It is likely that the results of this study are inconsistent with this prior evidence because of variations in the real-world use of smartphones among a population without consistent access to electricity. Specifically, a strategy that participants used to preserve the smartphone battery was to power the smartphone off when it was not in use. As geofence technology relies on real-time transmission of data, it is likely that one reason entry notifications were not received was as the smartphone was turned off when the geofence entry occurred.

Despite findings that GPS technology is not reliable for real-time ED or hospital use data, overall, participants expressed positive views of GPS technology. Participants embraced the idea of GPS being used by health care and other service providers to locate them if needed and described feeling more secure with the knowledge they could be found. This is similar to findings by Liss et al [9], who found that high-risk primary care patients were willing to use GPS technology to facilitate care coordination. Findings by Liss et al [9] also align with prior work by Moczygemba et al [37], which indicate that clinicians and care managers are particularly interested in using mHealth for care coordination among high-risk patients and patients experiencing homelessness [9,13]. This is particularly important as community health paramedics indicated that 57% (4/7) of ED or hospital visits were likely nonemergent visits that could have been addressed in the outpatient setting. Collectively, these findings suggest the need for app development and refinement as the GPS location tracking apps that are currently in the market do not have face validity or the specific functionality needed for use in the health care setting.

There was a significant decrease in depression symptoms from baseline to exit, which aligns with the qualitative findings where participants reported improved well-being and an overall positive experience with the intervention at the study exit. In contrast, a 1-month, pre-post study of homeless young adults (aged 18-24 years) who participated in a remote mental health intervention, which included SMS text messaging, did not find a difference in depression symptoms [38]. This may be as it takes longer than 1 month to see a difference in depression symptoms, although this finding warrants further study. The results also indicate an improvement in medication adherence as measured by the ASK-12. These findings support the findings of Morawski et al [39], in which the use of a smartphone app resulted in improved medication adherence among patients with hypertension. Participants in this study viewed the daily email question regarding medication adherence as a helpful reminder that supported adherence. The data also suggest that participants used their smartphones as a self-management support tool by downloading specific medication adherence apps or by setting alarms to help with medication management. This use of the smartphone as a tool is also evidenced by overall high scores regarding acceptance of technology at baseline and exit.

Although there was no significant difference in social support between baseline and exit, the qualitative data suggest that the smartphone had an impact on participants’ social connections. Prior evidence clearly indicates that social support can have a protective influence on multiple health outcomes among people experiencing homelessness [40] and that mobile phones are critical for individuals experiencing homelessness to maintain social connectedness to family and friends [41]. Thus, measuring social support in future studies investigating mHealth interventions among people experiencing homelessness is important for ascertaining a holistic picture of the benefits of smartphone technology among the homeless population.

Overall, the participants rated care transitions from the ED or hospital to the community fairly high. However, the results suggest that specific aspects of transitions could be improved. For example, in the critical understanding domain, two items related to understanding what and how to manage health on discharge and one item related to medication side effects scored lower than the remaining domain items. Future studies could investigate adapting the mHealth intervention to provide targeted follow-up post-ED or hospital discharge as well as specific guidance related to medication side effects to maximize adherence and optimize outcomes. Furthermore, the care plan domain scored the lowest among the four domains. This further supports the need to adapt the intervention to provide two-way communication between people experiencing homelessness and service providers to ensure that needed follow-up care is received in a timely and accessible manner.

### Study Limitations

The findings of this study should be interpreted with caution because of the study’s limitations. Participants were recruited from one city in a large, southern state using convenience sampling; therefore, the generalizability of the findings is unknown. Furthermore, participants were recruited from community sites, which may have biased the results. The pre-post design is subject to bias, and as study participants were selected on the basis of their PHQ-9 score, it is possible that regression to the mean occurred for the depression symptom outcome. There were also baseline differences in PHQ-9 scores between the groups that did and did not complete the study (*t*_21_=–2.17; *P*=.02) with the group that did not complete the study having a higher mean score at baseline than the group that did finish. The small sample size, although sufficient for answering this study’s research questions, may further limit the generalizability of the findings.

### Future Directions

The findings from this study point to several directions for future research. First, based on participants’ responses to the daily email medication adherence message and their stated preferences for SMS text messages, a subsequent study tested an expanded SMS text messaging intervention. That study also included testing the use of remote location services preinstalled on the smartphone to locate participants during business hours. The findings also suggest that in addition to unlimited access to a smartphone, access to unlimited transportation can facilitate the ability of people experiencing homelessness to self-manage chronic illness. Thus, future research could investigate the impact of providing accessible transportation on health outcomes and use. Finally, because of the shortcomings of GPS technology in communicating real-time health care use information for people experiencing homelessness and as there is an operational HIE in the local area, future research investigating care coordination should incorporate the HIE to ensure transmission of objective use data. Qualitative findings also suggest that mHealth interventions, particularly unlimited access to a smartphone and bus pass transportation, have numerous benefits for well-being and the ability of people experiencing homelessness to meet social needs. These concepts need to be explored quantitatively in future studies. Furthermore, coupling access to a smartphone and transportation with health care programs should be pursued at a policy level for local programs [42,43].

### Conclusions

mHealth interventions are acceptable to people experiencing homelessness and positively affected depression symptoms and medication adherence. Objective data from the HIE provided more accurate ED and hospital use information compared with alerts relying on predefined geofences. Despite this, participants favorably viewed GPS technology, warranting further exploration of GPS technology as a tool for facilitating care coordination among people experiencing homelessness.

## Abbreviations

ASK-12: Adherence Starts with Knowledge-12
CTM: care transitions measure
ED: emergency department
HIE: health information exchange
mHealth: mobile health
PHQ-9: Patient Health Questionnaire-9
